# Evaluating multidimensional facets of the maternal experience after preterm birth

**DOI:** 10.1038/s41372-024-01865-y

**Published:** 2024-01-18

**Authors:** Grace C. Fitzallen, James N. Kirby, H. Gerry Taylor, Helen G. Liley, Samudragupta Bora

**Affiliations:** 1 School of Psychological Sciences, College of Health and Medicine, University of Tasmania, Launceston, TAS Australia; 2 School of Psychology, Faculty of Health and Behavioural Sciences, The University of Queensland, Brisbane, QLD Australia; 3 Mater Research Institute, Faculty of Medicine, The University of Queensland, Brisbane, QLD Australia; 4 Center for Biobehavioral Health, Abigail Wexner Research Institute at Nationwide Children’s Hospital, and Department of Pediatrics, The Ohio State University, Columbus, OH USA; 5 Department of Pediatrics, University Hospitals Rainbow Babies & Children’s Hospital, Case Western Reserve University School of Medicine, Cleveland, OH USA

**Keywords:** Risk factors, Outcomes research

## Abstract

**Objective:**

Describe self-relating (self-criticism, self-compassion) and parenting competence (satisfaction, self-efficacy) in mothers of children born preterm, and their associations with child characteristics, maternal sociodemographics at childbirth, and maternal concurrent well-being.

**Study design:**

The sample comprised 1926 biological mothers of 3- to18-year-old children born preterm with self-ratings on the standardized Forms of Self-Criticising/Attacking & Self-Reassuring Scale, Self-Compassion Scale, and Parenting Sense of Competence Scale.

**Results:**

Mothers of children in early childhood reported significantly (*p* < 0.05) lower self-compassion than in middle childhood and adolescence. They also reported significantly lower parenting satisfaction than mothers of adolescents and higher self-efficacy than their middle childhood counterparts. Maternal psychosocial well-being was most strongly associated with self-compassion, parenting satisfaction, and self-efficacy after accounting for maternal psychopathology, child gestation, and child age.

**Conclusion:**

Longer-term associations of preterm birth with maternal self-relating and parenting competence emphasize broadening the scope of neonatal follow-up services, extending beyond child neurodevelopmental surveillance and postpartum psychopathology screening.

Postpartum depression is well-recognized as one of the most frequent maternal psychological diagnoses after childbirth. A recent meta-analysis found that mothers face between 1.2 and 18.4 times the risk for depression after preterm birth relative to term birth [1]. Similar risks have been reported for anxiety and posttraumatic stress [2, 3], and even in the absence of psychopathology, mothers report suboptimal well-being. A recent synthesis of 30 quantitative, qualitative, and mixed-methods studies of maternal experiences after preterm birth identified themes of shock and horror, consuming guilt, pervasive anxiety and hypervigilance, emotional numbing, and intrusive thoughts of the event [4]. These findings are concerning as psychopathology and/or suboptimal well-being have cascading implications for parental outcomes, particularly disrupted parent-infant bonding and maternal sensitivity.

Along with maternal psychopathology, parenting behaviors described as specific, observable, child caregiving behaviors have been extensively investigated in the preterm birth population. Nonetheless, there is limited investigation of maternal intrapersonal experiences beyond this scope spanning cognitive and affective dimensions. For example, one of the most recent meta-analyses on parenting among parents of children born preterm identified only four studies examining parenting attitudes (intrapersonal dimension), in contrast to 23 studies evaluating parenting behavior (interpersonal dimension) [5]. This illustrates the shortcomings of existing preterm birth follow-up research regarding intrapersonal parenting experiences, a key determinant of parenting behavior [6]. Therefore, a multidimensional conceptualization of maternal experiences is needed to comprehensively characterize mothers’ unique and complex experiences after preterm birth, recognizing their dynamic nature over time.

A core facet of parenting experiences that remains unclear after preterm birth is self-relating, the way individuals relate to themselves through self-directed, internalized processes in the face of adversities. Self-criticism involves high levels of self-evaluation where negative judgment, blame, and/or attack are present [7], whereas self-compassion involves self-reassurance, understanding, and kindness [8]. While these contrasting self-relating styles are often associated, they are distinct constructs [9].

Consequently, more critical self-relating increases the risk for the development and maintenance of psychopathology [7, 10], parenting stress, reduced acceptance of the parenting role and associated challenges [11], and notably, maladaptive parenting behavior [12]. Further, self-compassionate relating is independently associated with more favorable parenting outcomes. A recent meta-analysis of 13 studies synthesizing the benefits of interventions aimed at increasing parental self-compassion found significant reductions in parental psychopathology, including depression, anxiety, and stress, as well as increases in mindfulness [13, 14]. In addition, greater self-compassion is associated with reduced negative emotional responses to adversities including guilt and shame [15], and more adaptive parenting behaviors [16]. Positive effects of self-compassion in parents of children with autism spectrum disorder, a common neurodevelopmental outcome associated with preterm birth [17], have been identified. These include less disappointment, more functional relationships and greater emotional connection to the child, as well as decreased perceptions of difficult child behavior [18]. Nonetheless, research on maternal self-relating in the aftermath of preterm birth is limited, with self-criticism remaining a peripheral, secondary outcome, and insufficient conceptualization and inadequate description of how maternal self-relating changes over time.

Another related dimension that has been insufficiently investigated after preterm birth is parenting sense of competence, described as the caregiver’s beliefs and perceptions about their ability to effectively engage in parenting tasks [19]. Parenting competence has implications for parental expectations and maladaptive parenting behavior [6, 20]. This is important for parents of children born preterm, as perceived loss of parental role and autonomy is consistently identified as a significant stressor associated with neonatal intensive care unit admission. Pivotal to the development of parenting competence are the affective domain of satisfaction (i.e., frustration, anxiety, motivation), and the instrumental domain of self-efficacy (i.e., self-perceived beliefs about successfully completing parenting tasks) [19, 21]. Parenting competence in the general population typically increases over time as mastery is gained through experience; however, among parents of children born preterm, mixed findings have been reported, partially accounted for by varying study designs, conceptualizations of parenting outcomes, and assessment instruments. For example, a prospective study investigating maternal confidence after preterm birth, defined as confidence in the ability to provide adequate infant care, found decreasing confidence between 3–4 and 6–8 weeks after preterm birth [22]. Contrastingly, a cross-sectional study investigating parenting competence and self-efficacy in parents (98% mothers) of preterm- and term-born children at <24 months corrected age found greater parenting competence in mothers of older children, with no differences for the dimension of self-efficacy by age [23].

While the current focus of early neonatal follow-up including maternal psychopathology and parenting after preterm birth is valuable, the short- and long-term evaluation of intrapersonal maternal experiences has remained neglected. As a result, there is limited understanding of the extent of self-relating and parenting competence, particularly regarding the dynamic nature of these experiences after adverse birthing experiences, such as preterm birth. Long-term evaluation of maternal experiences after preterm birth is further justified due to expected changes in caregiving demands. Specifically, there are well-documented challenges experienced by mothers of children born preterm in coping with child behavioral and emotional difficulties that typically occur between school age and late adolescence [17, 24]. Hence, using a cross-sectional study design, this study aimed to describe self-relating (self-criticism and self-compassion) and parenting competence (satisfaction and self-efficacy) in mothers of children born preterm stratified by their child’s chronological age (early childhood, middle childhood, and adolescence). Another aim was to examine associations of self-relating and parenting competence with child characteristics, maternal sociodemographics at childbirth, and maternal concurrent well-being.

## Methods

### Participants

Between October 2019 and February 2020, primary caregivers of 3- to 18-year-old children born preterm (<37 weeks gestation) were recruited through parent organizations, with screening and outcome questionnaires administered via a secure web-based portal. If participants were the primary caregiver of more than one child born preterm, they were asked to consider their youngest child born preterm. For this study, eligible participants were biological mothers aged 22–63 years at assessment, for whom English was their primary (not necessarily native) language, and who resided in Australia, Canada, New Zealand, United Kingdom/Ireland, or United States of America. Further, mothers were eligible only if they served as a primary caregiver of a child born preterm who had not been diagnosed with a chromosomal anomaly, fetal alcohol spectrum disorder, and/or developmental disability (IQ < 70) if tested. In addition, mothers had to report being “*confident*”, “*very confident*”, or “*extremely confident*” on a 5-point Likert scale about their child’s gestational age at birth. Child gestational information was obtained twice, initially during eligibility screening and subsequently during post-consent data collection, using two distinct response formats. Of respondents (*N* = 2595/3300 eligible; 79%), gestational age reporting was congruent for 97%, with the remaining 3% (*n* = 80/2595) excluded from all analyses. A further reason for exclusion was incomplete responses (*n* = 589/2595 [23%], including 511 participants with no primary outcomes data). Therefore, of mothers who consented to participate, 74% were included in the current study (*N* = 1926/2595). The final sample comprised 1926 biological mothers stratified into three groups corresponding to their child’s chronological age: early childhood (3–5 years, *n* = 865), middle childhood (6–9 years, *n* = 611), and adolescence (10–18 years, *n* = 450). Analysis of participants excluded and included in the current study demonstrated mothers who were excluded were  more likely to be younger (*p* = 0.001), with lower educational attainment (*p* < 0.001), living within a lower family sociodemographic household at childbirth (*p* < 0.001), with a preterm-born child of slightly older gestations (*p* = 0.01).

The American Association for Public Opinion Research Best Practices for Survey Research [25] informed the design and conduct of this study. The University of Queensland Human Research Ethics Committee approved the study protocol, and all participants provided informed consent.

### Measures

Intrapersonal maternal outcomes were self-reported at a single time point on three standardized instruments. After a comprehensive literature review, these tools were selected primarily due to cultural appropriateness and age- and domain-specificity for the study population.

*Self-criticism* was assessed using the 22-item Forms of Self-Criticising/Attacking & Self-Reassuring Scale [26]. Mothers indicated their agreement with statements on a 5-point Likert scale from “*Not at all like me*” to “*Extremely like me*”, with higher scores representing greater critical self-relating dialogue. This scale has strong construct validity ranging from 0.91 to 0.95 in clinical populations [27, 28].

*Self-compassion* was assessed using the 26-item Self-Compassion Scale [8]. Mothers indicated how often they experienced self-compassionate relating on a 5-point Likert scale from “*Almost never*” to “*Almost always*”. The total score comprises six subdomains and is represented as a grand mean. Higher scores represent a greater capacity for self-compassionate relating. This scale has satisfactory psychometric properties including robust construct, discriminant, and convergent validity [8, 29].

*Parenting satisfaction and self-efficacy* were assessed using the 17-item Parenting Sense of Competence Scale [30]. Mothers rated their agreement with item statements on a 6-point Likert scale from “*Strongly disagree*” to “*Strongly agree*”, with higher scores representing greater satisfaction and self-efficacy independently. This scale has moderate to strong construct, convergent, and discriminant validity, and factor analysis supports the satisfaction and self-efficacy domains as assessing distinct aspects of parenting competence [19, 31].

### Statistical analyses

Data analysis was completed in three stages using the IBM^®^ SPSS^®^ Statistics software version 24.0. First, baseline sample characteristics were examined using the one-way analysis of variance for continuous variables and the Chi-square test of independence for categorical variables. Second, analysis of covariance with planned contrasts were performed to examine linear associations between maternal intrapersonal outcomes and the three child chronological age groups, with early childhood as the referent group for the test of linear associations and Cohen’s *d* as the measure of effect size. Country of residence was not included as a covariate due to marginal to negligible associations with the outcomes. Total psychopathology screening score on the Depression Anxiety Stress Scales Short Form (DASS-21) was entered as a covariate due to established independent associations with maternal outcomes and to account for potential self-reporting bias at the time of the assessment. All reported associations were reanalyzed with child age as a continuous variable using partial correlations controlling for maternal psychopathology screening score.

Third, a series of hierarchical linear regression modeling was undertaken to identify key variables associated with maternal intrapersonal outcomes. Variables were entered into the model in four blocks: (1) covariates including child gestational age at birth, child chronological age at assessment, and maternal total psychopathology screening score; (2) child characteristics including sex (coded as 0 = female, 1 = male), multiple birth (0 = singleton, 1 = multiple), neonatal risk (0 = low, 1 = high [presence of one or more of the following conditions: confirmed neonatal infection, oxygen therapy at 36 weeks, severe brain injury or abnormality]), and developmental risk (0 = low [none or developmental diagnosis in one domain], 1 = high [presence of two or more developmental diagnoses across neurobehavioral, neurosensory, physical/chronic domains]); (3) maternal sociodemographic characteristics at childbirth including age, educational attainment (0 = high, 1 = low [high school graduate or below]), family socioeconomic status (0 = high, 1 = low [unemployed, unskilled, semi-skilled]), parenthood status (0 = single, 1 = partnered), and identification with ethnic/racial minority (0 = no, 1 = yes); and (4) maternal concurrent well-being assessed using the Mental Health Continuum Short Form. Variables were modeled using backward and forward approaches to identify the most parsimonious model, with variables entered as a continuous measure where possible to optimize statistical precision. A *p* < 0.10 criterion was used to select variables for inclusion in the initial model, and a *p* < 0.05 criterion to retain variables in the final fitted model.

## Results

Sample characteristics, as shown in Table 1, demonstrate that mothers of adolescents had significantly (*p* < 0.05) greater proportions of young motherhood, low educational attainment, and single parenthood at childbirth. Mothers of children in early childhood reported significantly greater concurrent total psychopathology screening scores. Furthermore, children born preterm in the early childhood group had a significantly lower proportion of confirmed neonatal infection.

**Table 1:** Characteristics of the Sample (*N* = 1926).

Characteristics, % (Numerator/Denominator)	Child Age Groups			*p*
	Early Childhood [*n* = 865]	Middle Childhood [*n* = 611]	Adolescence [*n* = 450]	
**Maternal childbirth**				
Maternal age, mean ± SD, years	32 ± 5	31 ± 5	30 ± 5	<0.001
Young motherhood [≤21 years]	1.16 (10/865)	2.45 (15/611)	4.44 (20/450)	0.001
Minority ethnicity/race	8.58 (74/862)	9.56 (58/607)	8.28 (37/447)	0.73
Low education [high school graduate or below]	8.90 (77/865)	13.09 (80/611)	18.22 (82/450)	<0.001
Low family socioeconomic status [unemployed, unskilled, semi-skilled]	14.45 (125/865)	18.33 (112/611)	18.89 (85/450)	0.05
Single parent family	4.28 (37/865)	5.73 (35/611)	8.00 (36/450)	0.02
**Maternal concurrent**				
Total psychopathology score, mean ± SD	25.11 ± 19.82	23.26 ± 18.54	21.68 ± 18.82	0.002
Total well-being score, mean ± SD	49.23 ± 13.58	50.51 ± 12.94	50.93 ± 13.29	0.03
**Child neonatal**				
Gestational age, mean ± SD, weeks	31 ± 4	30 ± 4	30 ± 4	0.12
Extremely preterm, <28 weeks	23.01 (199/865)	25.70 (157/611)	24.45 (110/450)	0.42
Very preterm, 28– < 32 weeks	32.49 (281/865)	34.04 (208/611)	35.33 (159/450)	
Moderate/late preterm, 32– < 37 weeks	44.50 (385/865)	40.26 (246/611)	40.22 (181/450)	
Birthweight, mean ± SD, grams	1542 ± 681	1500 ± 684	1497 ± 657	0.25
Male sex	53.76 (465/865)	55.16 (337/611)	52.67 (237/450)	0.72
Multiple birth	30.87 (267/865)	32.24 (197/611)	32.22 (145/450)	0.81
Confirmed neonatal infection	24.39 (200/820)	30.12 (175/581)	29.83 (125/419)	0.03
Oxygen therapy at 36 weeks	36.27 (309/852)	41.57 (249/599)	40.99 (182/444)	0.08
Severe brain injury or abnormality	6.32 (54/855)	5.79 (35/604)	7.00 (31/443)	0.73
High neonatal risk^a^	45.43 (393/865)	53.52 (327/611)	54.89 (247/450)	0.001
**Child concurrent**				
Chronological age, mean ± SD, years	4 ± 1	7 ± 1	13 ± 2	<0.001
High developmental risk^b^	9.36 (81/865)	10.47 (64/611)	11.56 (52/450)	0.45
Country of residence				
Australia	36.42 (315/865)	35.02 (214/611)	37.33 (168/450)	0.16
Canada	15.38 (133/865)	12.44 (76/611)	10.67 (48/450)	
New Zealand	17.80 (154/865)	22.26 (136/611)	21.11 (95/450)	
United Kingdom/Ireland	15.26 (132/865)	13.42 (82/611)	15.78 (71/450)	
United States of America	15.14 (131/865)	16.86 (103/611)	15.11 (68/450)	

^a^Presence of one or more of the following conditions: confirmed neonatal infection, oxygen therapy at 36 weeks, severe brain injury or abnormality; 4% (*n* = 79/1926) participants with missing data were assigned to low neonatal risk group.

^b^Presence of two or more developmental diagnoses across neurobehavioral, neurosensory, physical/chronic domains.

### Maternal intrapersonal outcomes by child chronological age groups

As described in Table 2, analyses of maternal self-relating and parenting competence outcomes across the three child chronological age groups showed that mothers of children in early childhood reported significantly lower self-compassion than mothers of children in middle childhood (Cohen’s *d* = 0.11, *p* = 0.04) and adolescence (Cohen’s *d* = 0.25, *p* = 0.003). They also reported significantly lower parenting satisfaction than mothers of adolescents (Cohen’s *d* = 0.20, *p* = 0.001), and higher self-efficacy than mothers of children in middle childhood (Cohen’s *d* = 0.12, *p* = 0.02). No statistically significant associations were evident for the remaining pairwise comparisons. Similar patterns of reported associations were observed after reanalysis of the data with child age as a continuous variable (see Online Supplement).

**Table 2:** Associations Between Child Age Groups and Maternal Intrapersonal Outcomes, Accounting for Maternal Psychopathology (*N* = 1926).

Characteristics, Mean ± SD	Child Age Groups			Overall Linear *p*	Cohen’s *d*	
	Early Childhood [*n* = 865]	Middle Childhood [*n* = 611]	Adolescence [*n* = 450]		Early Childhood vs. Middle Childhood	Early Childhood vs. Adolescence
**Self-relating**						
Self-criticism	37.33 ± 6.71	36.84 ± 6.70	36.59 ± 6.70	0.13	0.07	0.11
Self-compassion	3.06 ± 0.56	3.12 ± 0.54	3.16 ± 0.55	0.007	0.11*	0.25**
**Parenting competence**						
Satisfaction	35.84 ± 6.59	36.25 ± 6.58	37.13 ± 6.60	0.003	0.06	0.20**
Self-efficacy	36.68 ± 5.85	35.98 ± 5.86	36.15 ± 5.88	0.06	0.12*	0.09

*p < 0.05, **p < 0.01

### Correlates of maternal intrapersonal outcomes

As shown in Table 3, higher maternal self-criticism was associated with younger maternal age (β = −0.07, *p* < 0.001) and lower family socioeconomic status (β = −0.07, *p* = 0.001) at the time of childbirth. Lower maternal self-compassion was also associated with younger maternal age at childbirth (β = 0.05, *p* = 0.001), as well as two-parent family at childbirth (β = 0.04, *p* = 0.02) and lower concurrent well-being (β = 0.40, *p* < 0.001). The final fitted regression models explained 31% and 51% of the variance in self-criticism and self-compassion, respectively.

**Table 3:** Fitted Regression Models for Correlates of Maternal Self-Relating Outcomes, Controlling for Child Gestation, Child Age, and Maternal Psychopathology.

Characteristics	Model 1: Covariates		Model 2: Child		Model 3: Maternal at Childbirth		Model 4: Maternal Concurrent	
	β	*p*	β	*p*	β	*p*	β	*p*
**Self-criticism**	**(*R***^**2**^** = 0.31,*p*** **< 0.001)**		**(*R***^**2**^** = 0.31,*p*** **< 0.001)**		**(*R***^**2**^** = 0.31,*p*** **< 0.001)**		**(*R***^**2**^** = 0.31,*p*** **< 0.001)**	
Child gestation	−0.01	0.46	−0.01	0.46	−0.01	0.47	−0.01	0.47
Child age	−0.04	0.04	−0.04	0.04	−0.05	0.02	−0.05	0.02
Maternal psychopathology	0.55	<0.001	0.55	<0.001	0.55	<0.001	0.55	<0.001
Maternal age	–	–	–	–	−0.07	<0.001	−0.07	<0.001
Low family socioeconomic status	–	–	–	–	−0.07	0.001	−0.07	0.001
**Self-compassion**	**(*R***^**2**^ = **0.41,** ***p*** < **0.001)**		**(*R***^**2**^ = **0.41,** ***p*** < **0.001)**		**(*R***^**2**^ = **0.42,** ***p*** < **0.001)**		**(*R***^**2**^ = **0.51,** ***p*** < **0.001)**	
Child gestation	0.01	0.44	0.01	0.44	0.01	0.46	−0.002	0.90
Child age	0.05	0.003	0.05	0.003	0.06	<0.001	0.05	0.002
Maternal psychopathology	−0.64	<0.001	−0.64	<0.001	−0.63	<0.001	−0.38	<0.001
Maternal age	–	–	–	–	0.06	0.002	0.05	0.001
Low maternal education	–	–	–	–	−0.05	0.004	–	–
Single parent family	–	–	–	–	0.04	0.04	0.04	0.02
Maternal well-being	−	–	–	–	–	–	0.40	<0.001

Additional variables entered and removed from model 2: sex, multiple birth, neonatal risk, and developmental risk; model 3: educational attainment and parenthood status (self-criticism only), family socioeconomic status (self-compassion only), and ethnic/racial minority; and model 4: maternal well-being (self-criticism only). “-”, variables not entered or removed from the model; β, standardized regression coefficient.

As shown in Table 4, lower maternal parenting satisfaction was associated with multiple birth status (β = −0.08, *p* < 0.001) and lower maternal concurrent well-being (β = 0.24, *p* < 0.001). Lower self-efficacy was also associated with multiple birth status (β = −0.08, *p* < 0.001), along with the absence of child developmental diagnosis or diagnosis restricted to a single domain (β = 0.04, *p* = 0.04), older maternal age at childbirth (β = −0.07, *p* = 0.001), and lower maternal concurrent well-being (β = 0.24, *p* < 0.001). The final fitted regression models explained 30% and 15% of the variance in parenting satisfaction and self-efficacy, respectively.

**Table 4:** Fitted Regression Models for Correlates of Maternal Parenting Competence Outcomes, Controlling for Child Gestation, Child Age, and Maternal Psychopathology.

Characteristics	Model 1: Covariates		Model 2: Child		Model 3: Maternal at Childbirth		Model 4: Maternal Concurrent	
	β	*p*	β	*p*	β	β	*p*	β
**Satisfaction**	**(*R***^**2**^ = **0.26,** ***p*** < **0.001)**		**(*R***^**2**^ = **0.27,** ***p*** < **0.001)**		**(*R***^**2**^ = **0.27,** ***p*** < **0.001)**		**(*R***^**2**^ = **0.30,** ***p*** < **0.001)**	
Child gestation	−0.01	0.55	−0.001	0.95	<0.001	0.99	−0.01	0.56
Child age	0.07	<0.001	0.07	<0.001	0.08	<0.001	0.07	<0.001
Maternal psychopathology	−0.50	<0.001	−0.50	<.001	−0.49	<0.001	−0.35	<0.001
Multiple birth	–	–	−0.07	<0.001	−0.08	<0.001	−0.08	<0.001
Maternal age	–	–	–	–	−0.04	0.06	–	–
Low family socioeconomic status	–	–	–	–	−0.05	0.04	–	–
Maternal well-being	–	–	–	–	–	–	0.24	<0.001
**Self-efficacy**	**(*R***^**2**^ = **0.11,** ***p*** < **0.001)**		**(*R***^**2**^ = **0.11,** ***p*** < **0.001)**		**(*R***^**2**^ = **0.12,** ***p*** < **0.001)**		**(*R***^**2**^ = **0.15,** ***p*** < **0.001)**	
Child gestation	−0.05	0.03	−0.03	0.20	−0.03	0.14	−0.04	0.08
Child age	−0.03	0.16	−0.03	0.14	−0.04	0.06	−0.04	0.04
Maternal psychopathology	−0.32	<0.001	−0.33	<0.001	−0.33	<0.001	−0.18	<0.001
Multiple birth	–	–	−0.09	<0.001	−0.08	<0.001	−0.08	<0.001
Developmental risk	–	–	0.05	0.03	0.05	0.03	0.04	0.04
Maternal age	–	–	–	–	−0.06	0.006	−0.07	0.001
Ethnic/racial minority	–	–	–	–	−0.04	0.09	–	–
Maternal well-being	–	–	–	–	–	–	0.24	<0.001

Additional variables entered and removed from model 2: sex, neonatal risk, and developmental risk (satisfaction only); model 3: parenthood status, ethnic/racial minority (satisfaction only), and family socioeconomic status (self-efficacy only). “-”, variables not entered or removed from the model; β, standardized regression coefficient.

## Discussion

This study, based on one of the largest samples to examine maternal outcomes after preterm birth to date, is novel in its multidimensional conceptualization and evaluation of long-term maternal intrapersonal experiences following preterm birth. Specifically, our research extends beyond the hospital stay and early childhood period, along with expanding the scope of investigation on postpartum psychopathology and parenting behavior outcomes, typical across follow-up studies of preterm birth. Study findings show mothers of children in early childhood reported lower self-compassion and parenting satisfaction compared with mothers of older children or adolescents, suggesting that these markers of suboptimal parental well-being following preterm birth are dynamic in nature, and may improve over time, at least at the group level. Nonetheless, we found similar reports of self-criticism among the three groups of mothers of children born preterm stratified by their child’s chronological age. Based on literature describing the acquisition of self-efficacy by Bandura (1982) [32] it was predicted that mothers of older children would report greater levels of self-efficacy in engaging with parenting tasks than mothers of younger children. Contrary to this prediction, mothers of children in early childhood (3–5 years of age) reported greater self-efficacy than mothers of children in middle childhood (6–9 years). It is plausible that this finding could be explained by the emergence of child behavioral and emotional difficulties during this period [17, 24], as well as an increase in the need and utilization of allied health support for a range of child-related challenges across domains of cognition, motor, language, and education [33].

While previous longitudinal research demonstrates long-standing and pervasive maternal psychopathology after preterm birth [34], findings of this study are consistent with parental quality of life converging closer to that of term birth over time [35]. In addition, current findings align with well-documented parenting research that describes the preschool-to-school entry transition period as one of the most stressful intervals for familial outcomes due to changes in routine, care responsibilities, child-related demands, and increased opportunity for parental comparisons. Among parents of children born preterm, these challenges are likely exacerbated due to the increased risk for poorer school readiness [36] and emerging child behavioral and emotional difficulties relative to their term-born peers [17]. Importantly, the findings of this study have identified a range of risk factors including child neonatal and sociodemographic characteristics. This information can be used for antenatal screening and targeted postpartum follow-up services.

Our findings provide support for the revised process model of parental functioning by Taraban & Shaw (2018) [37, 38], which highlights the importance of cognitive and affective parent characteristics in the development of optimal functioning. Further, findings highlight the importance of neonatal and early social factors in the development of these processes. This emphasizes the importance of early surveillance for mothers after preterm birth who may face a greater risk for disruptions in self-relating and parenting competence, particularly younger mothers, those who had a multiple birth pregnancy, and those who may not have a strong support system. Findings accentuate the unaddressed need for targeted support for intrapersonal outcomes beyond the treatment of early postpartum psychopathology and parenting behavior difficulties.

Our findings highlight the importance of a holistic and long-term characterization of maternal experiences after preterm birth [39–41]. This recommendation acknowledges the dynamic nature of parenting over time and will enable preventive approaches towards the inclusive optimization of outcomes for all parents rather than the reactive treatment of clinically significant cases. While several associations identified in this study are difficult to modify due to their complex nature, maternal psychosocial well-being and other related indicators (i.e., guilt, shame) may be more readily optimized through empirically supported psychosocial interventions. This includes but is not limited to compassion-focused therapy [42], compassionate mind training [43], acceptance and commitment therapy [44], mindfulness-based cognitive therapy [45], and the preterm birth-specific PREM-baby Triple P [46]. Despite the unique therapeutic differences of these approaches, collectively, they focus on intrapersonal outcomes and provide specific strategies and mechanistic pathways towards assisting parents who experience guilt and shame following a preterm birth.

Recently, due to technological advancements, psychosocial support has become more readily accessible through telehealth and social media avenues [47]. This is particularly pertinent for parents of children born preterm who may experience increased time demands because of ongoing child healthcare needs such as neurodevelopmental follow-up, relative to families with term-born children. Research has identified that mothers who use social media engage with a high level of information-seeking and sharing concerning their parenting role and consider internet-based connections as positive sources of social support [48]. The high proportion of families experiencing preterm birth who participate in neonatal and high-risk birth social media groups and pages, which were used to recruit for this study, supports the relevance of internet-based delivery systems for this population. While this aided in obtaining a large sample, these online recruitment efforts may have introduced selection bias to this study. Specifically, each organization had the primary aim of providing support and enhancing parental belonging. There are many benefits to receiving social support, particularly for mothers including reduced parenting stress [49], increased positive attitudes about being a mother [50], promoting adaptive and responsive parent-child interactions [51], and of note, buffering the negative association between parenting stress and satisfaction [52]. It is therefore plausible that our sample of mothers may have experienced a higher level of optimal well-being compared with mothers who do not belong to or relate with parenting organizations, potentially magnifying the importance of concurrent well-being as a key correlate for maternal outcomes.

There are additional limitations to our study that should be acknowledged while interpreting the findings. Despite extensive recruitment efforts, our sample predominantly comprised certain sociodemographic groups of mothers, including those of majority ethnicity/race (91%), high educational attainment (88%), and high family socioeconomic status (83%) at childbirth. While this study is novel in its multidimensional conceptualization of longer-term maternal intrapersonal experiences following preterm birth, caution should be exercised when considering these outcomes within the context of a more diverse and representative framework. Further, our sample was restricted to selected high-income countries with relatively homogenous parenting practices and access to advanced neonatal care with some form of maternal follow-up after preterm birth. There was also moderate potential for selection bias due to the requirement for English language fluency or access to a web-based device to complete the questionnaires. Other limitations include the cross-sectional study design, which did not allow the individual participant trajectories of the outcomes to be followed over time; and the psychometric quality of the available instruments for these outcomes in the context of the current population, which precluded comparison against standardized groups of parents. The potential impacts of these issues on the results are uncertain. Nevertheless, they identify pertinent areas for future research using prospectively recruited, longitudinally followed samples, representative of all infants born preterm and their families, particularly across factors including but not limited to diverse cultural and ethnic/racial backgrounds, socially determined parenting practices, and those residing in low- and middle-income countries. Furthermore, the findings from this study provide support for the continued investigation of multidimensional parenting outcomes after high-risk birth, with future research encouraged to include term-born comparison groups and an inclusive approach to parental recruitment.

In conclusion, study findings support adopting a multidimensional conceptualization of maternal experiences after preterm birth, extending beyond the current focus on postpartum psychopathology and parenting behavior. Further, findings highlight the need for a holistic, longer-term, psychosocial well-being framework of screening and intervention to optimize both maternal and child outcomes.

### Supplementary information

Online Supplement

## Data Availability

The corresponding author will make data from this study available upon reasonable request.
