# Supplementary material for: Evaluating multidimensional facets of the maternal experience after preterm birth
Source: J Perinatol. 2024 Jan 18;44(5):635–42. doi: 10.1038/s41372-024-01865-y (PMC11090785; doi:10.1038/s41372-024-01865-y)

Online Supplement: Associations Between Child Chronological Age and Maternal Intrapersonal Outcomes Following Preterm Birth.

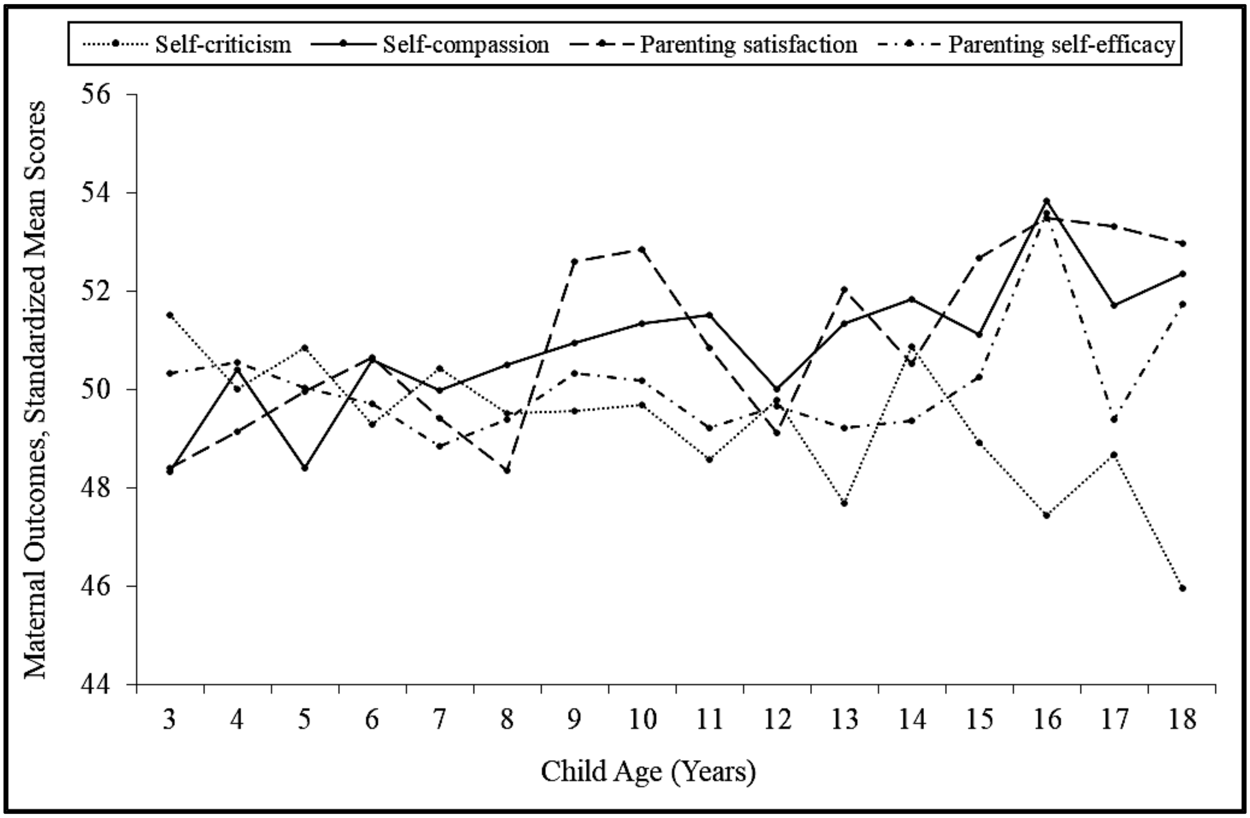

Supplement: Supplementary file 1 — Online Supplement [file 41372_2024_1865_MOESM1_ESM.pdf]
